# Adsorption and diffusion characteristics of lithium on hydrogenated α- and β-silicene

**DOI:** 10.3762/bjnano.8.175

**Published:** 2017-08-23

**Authors:** Fadil Iyikanat, Ali Kandemir, Cihan Bacaksiz, Hasan Sahin

**Affiliations:** 1 Department of Physics, Izmir Institute of Technology, 35430, Izmir, Turkey; 2 Department of Materials Science and Engineering, Izmir Institute of Technology, 35430, Izmir, Turkey; 3 Department of Photonics, Izmir Institute of Technology, 35430, Izmir, Turkey; 4 ICTP-ECAR Eurasian Center for Advanced Research, Izmir Institute of Technology, 35430, Izmir, Turkey

**Keywords:** density functional theory, diffusion, Li atom, silicene, ultra-thin materials

## Abstract

Using first-principles density functional theory calculations, we investigate adsorption properties and the diffusion mechanism of a Li atom on hydrogenated single-layer α- and β-silicene on a Ag(111) surface. It is found that a Li atom binds strongly on the surfaces of both α- and β-silicene, and it forms an ionic bond through the transfer of charge from the adsorbed atom to the surface. The binding energies of a Li atom on these surfaces are very similar. However, the diffusion barrier of a Li atom on H-α-Si is much higher than that on H-β-Si. The energy surface calculations show that a Li atom does not prefer to bind in the vicinity of the hydrogenated upper-Si atoms. Strong interaction between Li atoms and hydrogenated silicene phases and low diffusion barriers show that α- and β-silicene are promising platforms for Li-storage applications.

## Introduction

Following the first synthesis of graphene, the family of two-dimensional (2D) materials have drawn extraordinary attention [1–2]. This family consists of a large variety of materials such as hexagonal boron nitride (hBN) [3–4], silicene [5–7], germanene [8], transition-metal dichalcogenides (TMDs) [9–14], transition-metal trichalcogenides (TMTs) [15–16], phosphorene [17] and gallium chalcogenides [18]. Structural stability, chemical versatility and electronic band gaps of 2D materials that cover the range from 0 to 5 eV make them attractive for current nanoscale device applications.

In the large family of 2D materials, silicene deserves a special consideration due to its compatibility and expected integration with current nanotechnology. Silicene consists of a single layer of Si atoms arranged in a hexagonal lattice. Unlike the gapless semimetal graphene, silicene has a tiny energy gap that stems from the intrinsic spin–orbit interaction [19]. Instead of the planar structure of graphene, silicene exhibits a low-buckled structure.

Although bulk silicon does not have a layered structure, syntheses of a 2D form of silicon via epitaxial growth on several metal substrates such as Ag(111) [5,20], Ir(111) [21], and ZrB_2_(0001) [22] were achieved. By performing ab initio calculations, Liu et al. predicted that the electronic properties of silicene highly depend on the substrate [23]. Johnson et al. showed that the Ag(111) surface leads to metalization of a few distinct forms of silicene [24]. Among the variety of substrates, Ag(111) surface comes to prominence for epitaxial growth of single-layer silicene. Lattice match and almost homogeneous interaction between Ag(111) and silicene support the formation of a honeycomb structure of silicene. Recently, a silicene field-effect transistor was successfully fabricated on Ag(111) with a measured room-temperature mobility of about 100 cm^2^·V^−1^·s^−1^ [25]. In addition to pristine forms, hydrogenated derivatives of silicene were also studied extensively. Theoretically, it was predicted that hydrogenated silicene has two different atomic configurations (chair-like and boat-like) with energy gap values ranging between 2.9 and 4.0 eV [26]. It was found that half-hydrogenated silicene exhibits ferromagnetic semiconducting behavior with a band gap of 0.95 eV [27]. Hydrogenation leads indirect-to-direct gap transitions in bilayer silicene [28]. In the experimental study of Qiu et al., the ordered and reversible hydrogenation of silicene was performed [29]. Moreover, Medina et al. demonstrated that hydrogenation leads to a structural transition from the classical α-(3×3) phase to the β-(3×3) phase [30]. It was indicated that β-(3×3) phase could coexist with α-(3×3) phase. Despite recent experimental studies on these phases, no theoretical study has ever been reported for the hydrogenated forms of α- and β-silicene on a Ag(111) surface.

The adsorption of alkali metal atoms provides various ways to modify the structural, electronic and magnetic properties of 2D materials. It was found that adsorption of alkali atoms is a proper way to dope carbon nanotubes chemically [31–32]. It was reported that the hydrogen storage capacity and conductivity of single-walled carbon nanotubes could be enhanced by doping with Li and K [33]. The adsorption of Li atoms on the graphene surface was extensively studied [34–36]. It was found that the interaction of alkali metal atoms with silicene is stronger than with graphene, and the adsorption of metal atoms leads to the metalization of silicene [37]. It was calculated that the adsorption of Li atoms results in the stabilization of the unstable distorted T-phase of MoS_2_ [38]. In addition, Zr-based MXenes were found to be candidates as electrode materials for Li-ion batteries [39]. For applications in Li-ion batteries, a high coverage of Li atoms on a material is required. Due to its buckled large surface area, silicene seems to be a good candidate for Li-ion battery applications. Li adsorption on pristine silicene has been extensively studied in the last several years [40–46]. To the best of our knowledge, the adsorption characteristics of a Li atom on hydrogenated silicene are still unknown.

In the present paper, we study the diffusion and adsorption characteristics of a Li atom on recently synthesized hydrogenated forms of α- and β-silicene phases on a Ag(111) surface using ab initio calculations within density functional theory. The paper is organized as follows: Computational methodology, hydrogenated structures of the silicene phases on Ag(111) surface, and the diffusion and adsorption characteristics of Li are presented in the section “Results and Discussion”. Lastly, we conclude our results in section “Conclusion”.

## Results and Discussion

### Computational methodology

The present calculations were performed using density functional theory (DFT) and the projector-augmented wave (PAW) method, as implemented in the “Vienna ab initio Simulation Package” (VASP) [47–48]. The exchange–correlation energy was described by the generalized gradient approximation (GGA) using the Perdew–Burke–Ernzerhof (PBE) functional [49]. A plane-wave basis set with kinetic energy cutoff of 500 eV was used for all the calculations. The van der Waals (vdW) correction to the GGA functional was included by using the DFT-D2 method of Grimme [50].

To properly simulate the structures, a 3×3-reconstructed hydrogenated silicene phase was placed on top of a 4×4 supercell of a two-layer Ag(111) surface. A 3×3×1 Γ-centered *k*-point mesh was used for the Brillouin zone integration. The cohesive energy per atom was formulated as

[1]
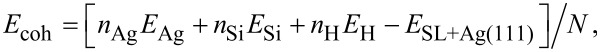


where *E*_Ag_, *E*_Si_ and *E*_H_ denote the single-atom energy of atoms Ag, Si and H, respectively. *n*_Ag_, *n*_Si_ and *n*_H_ are the number of Ag, Si, and H atoms contained in the unit cell, respectively. *E*_SL+Ag(111)_ denotes the total energy of single-layer hydrogenated silicene and two-layer Ag(111). *N* is the number of total atoms contained in the unit cell.

Binding energies were calculated for the most favorable adsorption sites. Binding energies of the Li atom were calculated by using the formula

[2]
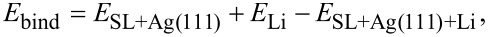


where *E*_bind_ is the binding energy of Li atom on the hydrogenated α- or β-silicene, *E*_SL+Ag(111)_ is the energy of hydrogenated α- or β-silicene on a two-layer Ag(111) surface, *E*_Li_ denotes the energy of a single isolated Li atom, and *E*_SL+Ag(111)+Li_ is the total energy of Li atom, single-layer hydrogenated silicene and two-layer Ag(111) surface.

Lattice constants and total energies were computed using the conjugate-gradient algorithm. The total energy difference between the sequential steps in the iterations was taken to be 10^−5^ eV for the convergence criterion. The total force in the unit cell was reduced to a value of less than 10^−4^ eV/Å. To hinder interactions between the adjacent cells, at least 12 Å vacuum space was used along the *z*-direction. All calculations were performed taking into account the spin-polarized case. Analysis of the charge transfers in the structures was determined by the Bader technique [51].

### Hydrogenated α- and β-silicene on a Ag(111) surface

Scanning tunneling microscopy (STM) measurements revealed that hydrogenated silicene on Ag(111) surfaces exhibits two different perfectly ordered phases, which are hydrogenated α-(3×3)-silicene and hydrogenated β-(3×3)-silicene [30]. For simplicity, we name the hydrogenated α- and β-phases “H-α-Si” and “H-β-Si”, respectively. Due to the existence of the Ag(111) surface, one side of silicene is accessible to hydrogenation. Previous studies showed that H atoms interact with the upper Si atoms of silicene and enhances the buckling of H-α-Si and H-β-Si [30]. Therefore, the structures of H-α-Si and H-β-Si do not exhibit significant differences to their pristine counterparts.

To determine how many layers of Ag (111) correctly simulate the supported α- and β-surfaces we examined the structural and electronic properties of these two phases on both two-layer and four-layer Ag(111) surfaces. As shown in Table 1, charge distribution and atomic distances are almost the same for both two layers and four layers. Therefore, it is reasonable to assume that characteristic properties of Ag(111)-supported silicene can be well simulated using two layers of Ag(111).

**Table 1:** The calculated silicene–substrate distance *d*, thickness of silicene and valence charges on upper-Si (ρ_u_) and lower-Si (ρ_l_) atoms of α-Si and β-Si on two-layer and four-layer Ag(111) surfaces.

	*d*	thickness of silicene	ρ_u_/ρ_l_
	(Å)	(Å)	(e^−^)

α-Si/2L Ag(111)	2.40	0.80	3.9/4.0
α-Si/4L Ag(111)	2.41	0.77	3.9/4.0
β-Si/2L Ag(111)	2.40	0.67	3.9/4.0
β-Si/4L Ag(111)	2.42	0.70	3.9/4.0

**Figure 1:**
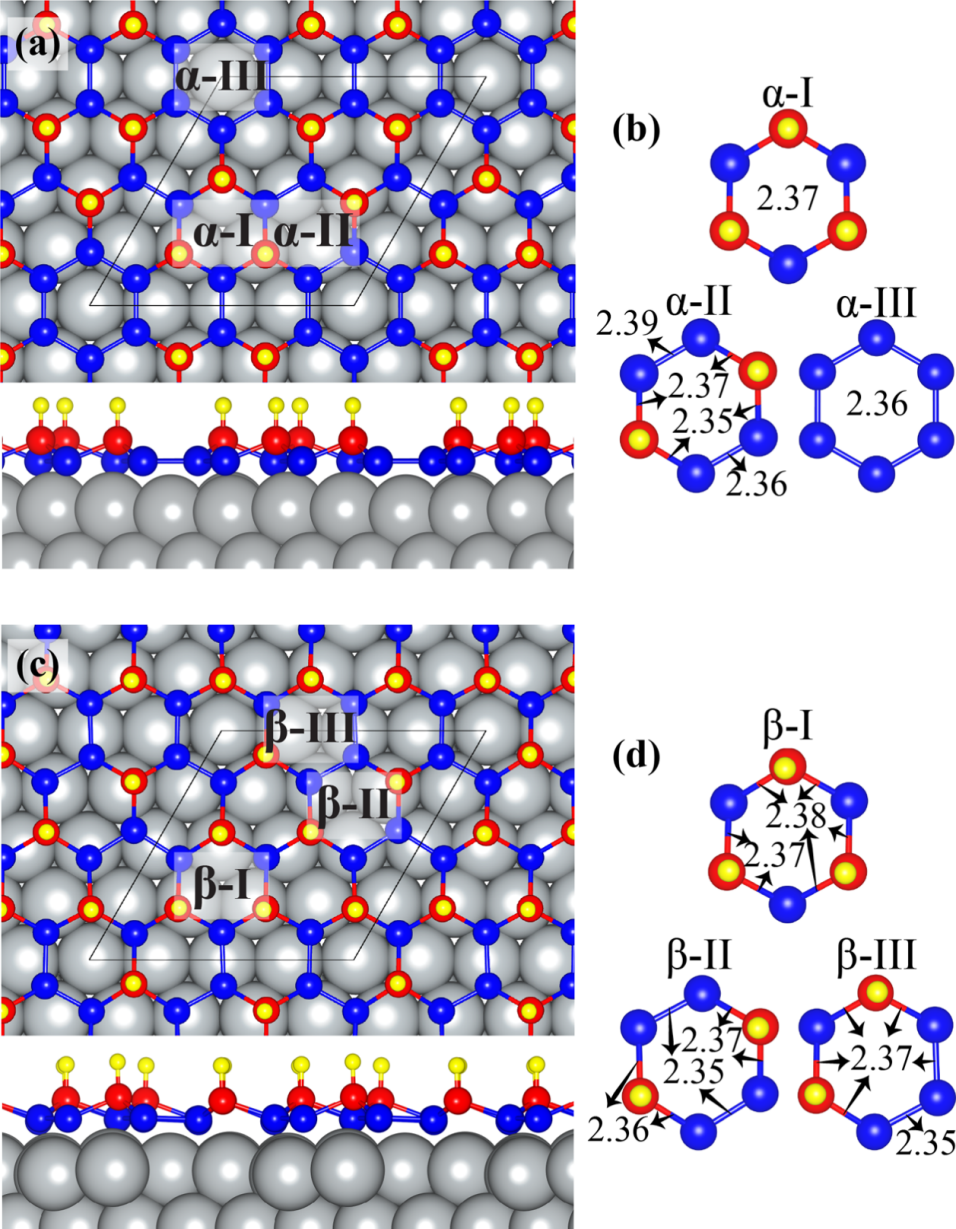
Top and side views of (a) H-α-Si and (b) its three different hexagonal units α-I, α-II, and α-III. Top and side views of (c) H-β-Si and (d) its three different hexagonal units β-I, β-II, and β-III. The 3×3 unitcells are represented by a black rhombus. Grey, blue, red, and yellow atoms show Ag, lower-Si, upper-Si, and H atoms, respectively.

Single layers of H-α-Si and H-β-Si are placed on two layers of a 4×4 supercell of Ag(111). While the bottom-layer atoms of Ag(111) are totally fixed, the upper-layer atoms are free to move. The optimized geometric structures of H-α-Si and H-β-Si on Ag(111) are shown in Figure 1a and Figure 1c, respectively. To better demonstrate the structures, the lower and upper Si atoms are shown by blue and red atoms, respectively. Grey and yellow atoms denote, respectively, Ag and H. As seen in Figure 1, H-α-Si and H-β-Si do not exhibit the conventional low-buckled structure of silicene. Our total energy calculations show that the cohesive energies of both phases are almost the same which indicates that both phases can exist at the same time. This is in good agreement with previous studies reporting the coexistence of α- and β-phases. Therefore, a detailed structural analysis of these two surfaces are important to clearly understand adsorption and diffusion characteristics of an adatom on these surfaces.

Hexagonal units in the H-α-Si possess three different configurations, such as α-I, α-II, and α-III, which are shown in Figure 1b. α-I is similar to the low-buckled structure of pristine silicene the nearest silicene atoms of which are lower Si and upper Si atoms. In this hexagonal unit, Si–Si bond distances are 2.37 Å. In α-II, there are two lower-Si-dimers that are placed opposite to each other. Si–Si bond lengths of two Si dimers are 2.36 Å and 2.39 Å. The bond distances between upper Si and lower Si atoms are 2.35 Å and 2.37 Å. α-III consists of six lower Si atoms. Si atoms in the α-III exhibit an almost planar structure with bond lengths of 2.36 Å. As a result, H-α-Si has six upper Si and twelve lower Si atoms. H atoms are placed at the top of the six upper Si atoms with a bond length of 1.51 Å. The cohesive energy of H-α-Si is 3.23 eV. The distance between lower Si atoms and the Ag(111) surface is about 2.07 Å.

Similar to H-α-Si, the H-β-Si structure also has three different hexagonal units, namely β-I, β-II, and β-III, which are shown in Figure 1d. β-I has a geometric structure similar to that of α-I. However, compared to α-I, the distances between lower Si and upper Si atoms are not the same and they are 2.37 Å and 2.38 Å. β-II has two lower-Si-dimers which are placed opposite to each other. Si–Si bond lengths of both lower Si dimers are 2.35 Å. The bond distances between upper Si and lower Si atoms are 2.36 Å to 2.37 Å. β-III has two neighboring lower Si dimers. Three Si atoms in these two neighboring Si dimers are almost in the same plane with bond lengths of 2.35 Å and 2.37 Å. The bond distances between upper Si and lower Si atoms are 2.37 Å. Consequently, unlike the H-α-Si, H-β-Si consists of seven upper Si and eleven lower Si atoms. H atoms are placed at the top of these seven upper Si atoms with a bond length of 1.51 Å. Because of the less symmetric atomic structure of H-β-Si the hexagonal units in H-β-Si are highly distorted. The cohesive energy of H-β-Si is 3.22 eV. The distance between lower Si atoms and the Ag(111) surface is about 2.16 Å. Therefore, one may expect quite different adsorption and diffusion characteristics of Li atoms on H-α-Si and H-β-Si.

### Diffusion of a Li atom on hydrogenated α- and β-silicene

To understand how a Li atom adsorbs and migrates on H-α-Si and H-β-Si surfaces, total energy calculations are performed by placing a Li atom on 13 different points, which include the high-symmetry points. The distance between Li atom and surface is fully relaxed while the position of the Li atom parallel to the surface is kept fixed. During the adsorption, while Li and the nearest atoms to Li are fully relaxed, rest of atoms in the unit cell are fixed. Diffusion barriers are determined by setting the total energy of the most favorable site to zero. The most favorable binding sites are determined and their binding site, binding energy, height on the surface, the amount of charge transfer and energy barriers are given in Table 2.

**Table 2:** The calculated ground-state properties of a Li atom on H-α-Si and H-β-Si: binding site, binding energy, distance from adsorbed Li atom to the surface of H atoms Δ*h*, the amount of charge donated by the Li atom Δρ, and the energy barrier (relative to the binding site).

	binding site	binding energy	Δ*h*	Δρ	energy barrier
		(eV)	(Å)	(e^−^)	(meV)

Li/H-α-Si	6Si	2.79	−1.19	−1	768
Li/H-β-Si	3HSi′	2.82	0.07	−1	411

**Figure 2:**
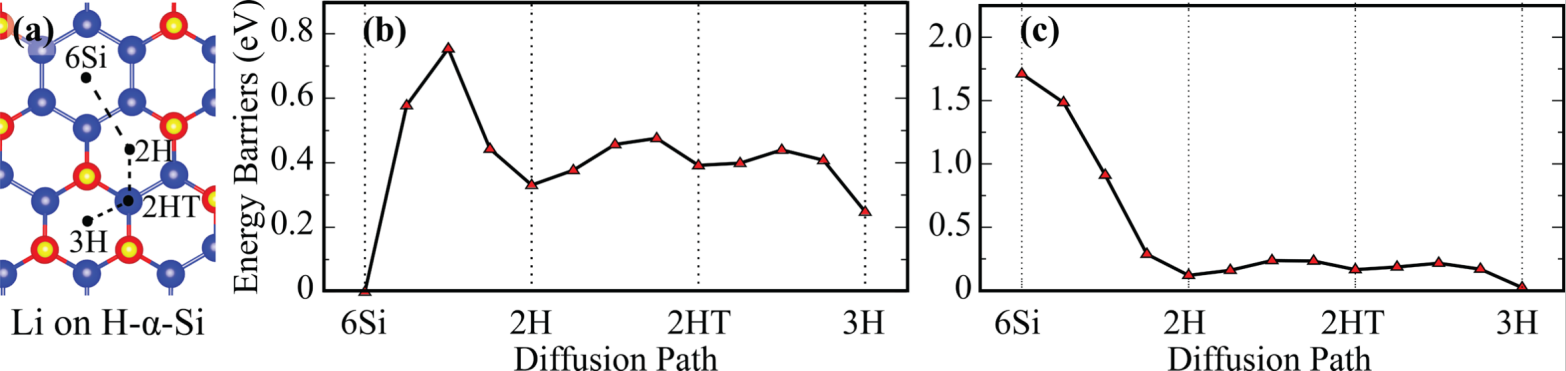
(a) Top view of possible adsorption sites for a Li atom on H-α-Si. Energy barrier graphs of (b) the first and (c) the second Li atom on H-α-Si. A possible diffusion path of a Li atom is illustrated by dashed black lines.

As shown in Figure 2a, four sites of 6Si, 2H, 2HT and 3H are considered as different binding sites for H-α-Si. Our calculations reveal that the most favorable site is 6Si, with the Si–Li bond length of 2.70 Å. In this binding site, Li atom binds with six lower Si atoms and the height of the Li atom is −1.19 Å lower than those of the H atoms. The binding energy of the Li atom on H-α-Si is 2.79 eV. Bader charge analysis shows that the Li atom donates 1e^−^ to H-α-Si. Since the Li atom does not prefer to bind to the vicinity of H atoms, the nearest adsorption site to 6Si is 2H. It is reasonable to assume that Li atoms diffuse through these two favorable adsorption sites. The diffusion barrier of a Li atom between these two nearest binding sites is 768 meV. However, once the Li atom overcomes the energy barrier, its diffusion through the other possible sites 2H, 2HT, and 3H (having energy barriers of 100–250 meV) is more likely. We also study diffusion of a second Li atom on H-α-Si and the energy barrier graph of it is shown in Figure 2c. The most favorable site of the second Li atom is 3H and the nearest adsorption site to 3H is 2HT. The diffusion energy barrier of the second Li atom between 3H and 2HT sites is 193 meV, which is 575 meV lower than that of the first Li atom. Hence, after the 6Si sites are occupied by the first Li atoms, the diffusion of the second Li atoms occur through the other sites relatively easily. As a result, despite such a large diffusion energy barrier between two most favorable sites, diffusion of Li atoms on H-α-Si is still possible.

**Figure 3:**
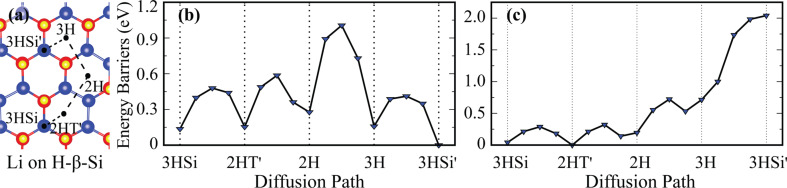
(a) Top view of possible adsorption sites for Li atom on H-β-Si. Energy barrier graphs of (b) the first and (c) the second Li atom on H-β-Si. A possible diffusion path of Li atom is illustrated by dashed black lines.

Five sites, namely 3HSi, 2HT′, 2H, 3H and 3HSi′ on H-β-Si are shown in Figure 3a. The most favorable site of a Li atom on this surface is 3HSi′. The Li atom is placed in the middle of three H atoms, and it is almost in the same plane with these H atoms. The bond distances from the Li atom to the three H atoms are 1.94 Å. Li atom binds to H-β-Si with a binding energy of 2.82 eV, which is ca. 30 meV higher than that on H-α-Si. Therefore, Li atoms bind to H-β-Si slightly more easier than to H-α-Si. On H-β-Si, the Li atoms forms an ionic bond and it donates 1 e^−^ to the surface. The nearest site to the most favorable site of 3HSi′ is 3H and this site is also energetically the second most favorable site. Thus, diffusion through these two favorable adsorption sites is most likely. The diffusion barrier of a Li atom on H-β-Si is 411 meV. Therefore, the energy barrier for the Li atom on H-β-Si is almost half of that on H-α-Si. As can be seen from Figure 3c, the 2HT′ site is the energetically most favorable site for the second Li atom on H-β-Si. The calculated diffusion energy barrier of the second Li atom between 2HT′ and 3HSi sites is 287 meV.

**Figure 4:**
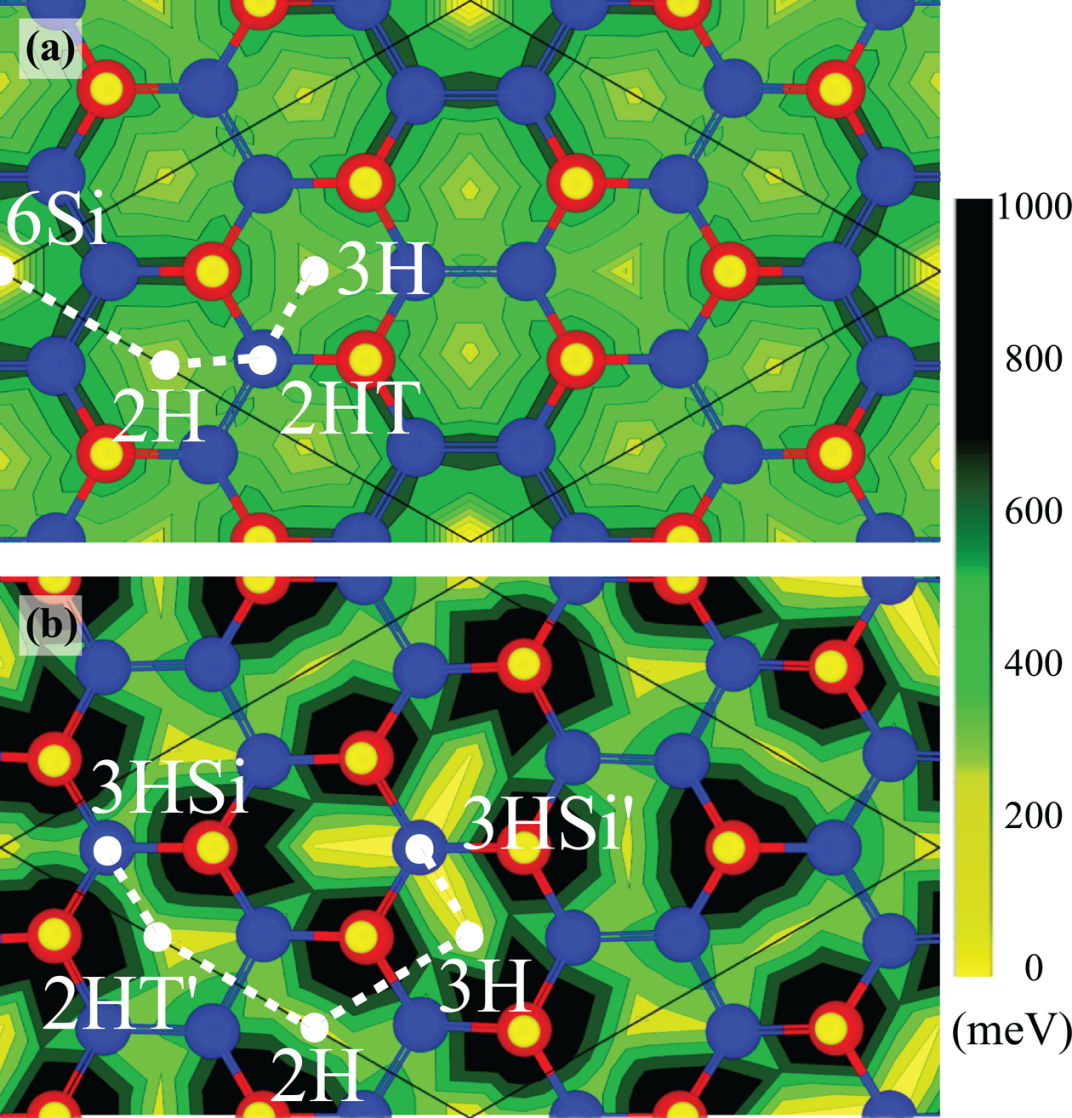
(Color online) Contour plots of the energy barriers (in meV) seen by Li atom adsorbed on (a) H-α-Si and (b) H-β-Si. Possible diffusion paths of Li on hydrogenated α and β silicene surfaces are illustrated by white dashed lines.

Contour plots of the energy barriers of a Li atom on H-α-Si and H-β-Si surfaces are shown in Figure 4a and Figure 4b, respectively. As seen from the figure, the energy differences for a Li atom on the H-β-Si surface are in a broader range than that on the H-α-Si surface. Diffusion of the Li atom around the favorable sites of H-β-Si is restricted because of high energy barriers around H atoms. In spite of the high diffusion barrier in H-α-Si, when Li atoms occupy all of the most favorable sites of 6Si, the following Li atoms on H-α-Si may diffuse more easily than on H-β-Si. The highest energy barriers seen by a Li atom are at the top of H atoms for both surfaces. These results suggest that a diffusing Li atom can follow a path of minimum energy barriers through the hydrogenated upper-Si atoms on both surfaces. In addition to the tops of the hydrogenated upper Si atoms, the tops of the lower Si atoms near to the 6Si site are also forbidden for Li atoms on H-α-Si.

## Conclusion

The adsorption properties and diffusion characteristics of a Li atom on single-layer hydrogenated silicene on a Ag(111) surface were investigated. Structural properties of recently synthesized fully hydrogenated α- and β-silicene phases were investigated in detail. Our results showed that a single Li atom forms a strong ionic bond with H-α-Si and H-β-Si surfaces. The Li atom prefers to bind to the 3HSi′ site of H-β-Si with a binding energy of 2.82 eV. Due to the high diffusion energy barrier, a single Li atom is trapped at the 6Si site of H-α-Si, with a binding energy of 2.79 eV. However, when all the 6Si sites of H-α-Si are occupied, the diffusion barriers seen by Li atom decreases. Possible pathways of various diffusion processes of a Li atom were studied in detail. It was found that the Li atom does not prefer to bind in the vicinity of the hydrogenated upper-Si atoms on H-α-Si and H-β-Si. It is worth mentioning that H-α-Si surface is dominated by moderate energy regions, whereas the H-β-Si surface consists of partially convenient or forbidden regions for Li atom diffusion. High binding energies and relatively low diffusion barriers for a Li atom on H-α-Si and H-β-Si suggest that hydrogenated forms of α- and β-silicene are suitable materials for Li-ion batteries.
